# Exploring young people’s views of their local area related to the 20-minute neighbourhood policy: a national cross-sectional study

**DOI:** 10.1080/23748834.2024.2315804

**Published:** 2024-02-29

**Authors:** Marissa Bulloch, Jonathan R. Olsen

**Affiliations:** a School of Health and Wellbeing, University of Glasgow, Glasgow, UK; b MRC/CSO Social and Public Health Sciences Unit, University of Glasgow, Glasgow, UK

**Keywords:** Young people, place standard, 20-minute neighbourhoods, inequalities, planning, health

## Abstract

This study aimed to examine how young people subjectively perceive their local neighbourhoods and compare these perceptions with objective data regarding access to amenities aligned with features of the 20-minute neighbourhood (20MN) policy. Objective data (n:287 aged 12-18 years) were gathered through an online adaptation of the Place Standard Tool for Young People in Scotland. Subjective data concerning access to ten amenities in line with the 20MN concept, along with crime statistics, area-level deprivation and urbanicity were spatially linked. The association between perceptions of localities and factors such as gender, as well as both subjective and objective neighbourhood assessments were examined. Young people were most satisfied with nature and active travel in their local area. Conversely, their satisfaction was lowest for active travel to school. Rural young people were more dissatisfied with their localities. Agreement was observed between satisfaction with their 20 neighbourhoods and objective data indicating the presence of frequent public transportation, recreational spaces, and access to services. In conclusion, our study highlights the importance of amenities in healthy urban planning, showing a positive impact on young people’s perceptions. Additionally, we uncover a connection between crime perceptions and area-level crime variables, potentially influencing health outcomes.

## Introduction

There is an extensive literature base to support the notion that our neighbourhood of residence can impact our health and overall quality of life through numerous pathways (Diez Roux 2001). For example, the environment where we live influences our ability to access services we need, be social, physically active, as well as feel safe. Governments worldwide, including the Scottish Government, recognised that ‘it is not primarily in our hospitals or our GP surgeries that health is first created. It is in our homes and our communities; in the places we live and through the lives we lead’ (Scottish Government 2018a). Place-planning therefore has a vital role in shaping our surroundings in a way that creates and maximises opportunities for healthy living. Young people often have their needs and requirements overlooked in place-planning due to priorities based on the adult population (Bishop and Corkery 2017). However, adolescence is a key period where public policy influences behaviours that can impact health prospects in later life (Raphael 2013).

In recent years, the idea of ‘local living’ has returned to the forefront of many national and local planning policies (Gower and Grodach 2022). The 20-minute neighbourhood is one method of local living that is receiving strong support from many governments worldwide, including Scotland (Scottish Government 2023). The aim of a 20-minute neighbourhood is to design places so that residents can reach most of their daily needs, such as shopping, healthcare, schools, and leisure activities, within a 20-minute round-trip from their home on foot. By ensuring everyone has equitable access to everything deemed essential for daily living, it has been proposed that this may help to reduce population level health inequalities, as well as having benefits on the climate and the local economy through reducing the need for motorised transport (O’ Gorman and Dillon-Robinson 2021). However, a Scottish study of neighbourhood accessibility within 20-minute neighbourhoods found that those living in the most deprived areas had the greatest access to essential facilities and services such as public transport, primary health care and accessible open space (Olsen *et al*. 2022). These findings suggest that objective data alone may not be the most informative metric to place-planners and policymakers when considering what is needed to improve local neighbourhoods and reinforces that the quality of these facilities should be considered in tandem.

It is vital that place-planning considers the individual needs of local areas by gaining context and insight of what a quality environment means to the local community. The Scottish government has recognised this in their fourth national planning framework (NPF4) (Scottish Government 2023), which encourages planning authorities to make use of the Place Standard Tool to adopt a collaborative approach with the community in future development planning. The Place Standard tool (Hasler 2018) is a survey tool that collects subjective data about the local environment by asking participants to think about the quality of their area through 14 questions encompassing aspects of both the physical and social environment. Since the tool’s development, it has been used to generate conversations about place in various parts of the world, including the UK (Hasler 2018), Spain (Ocaña *et al*. 2022), North Macedonia (Gjorgjev *et al*. 2020) and Cyprus (Kleopa *et al*. 2022). It has served the purpose of supporting planning and service delivery by local authorities, assessing municipalities with a focus on social determinants of health and equity across diverse settings. Additionally, the tool is used to stimulate community and stakeholder dialogue on the creation of healthy and equitable places. Given its success, versions for children and young people have also been developed (Our Place 2023). Specific tools designed for young people are important to provide an opportunity to raise their concerns about their community, increasing social cohesion, whilst also giving youth a sense of responsibility.

The local environment is important for young people, research has found links between young people’s own assessment of their neighbourhood and health outcomes. A favourable local environment was found to be positively associated with physical activity in a large sample of Spanish young people, where children who perceiving their environment more positively were more likely to meet the recommended daily level of physical activity (Garcia-Cervantes *et al*. 2016). The influence of the built environment and use of local spaces by young people has been shown to differ by age and gender (Kowaleski-Jones *et al*. 2017) and living in areas of higher socioeconomic disadvantage can influence perceptions of local facilities, such as greenspaces (Jones *et al*. 2009). Although there is little evidence of neighbourhood perceptions for young people by socioeconomic status, there is evidence that living in a more socioeconomic deprived area in childhood can influence the risk of non-communicable diseases in later life (Kivimäki *et al*. 2018). Gender differences have been reported in perceptions of place, the neighbourhood environment, independent mobility, and health (Stafford *et al*. 2005, Porter *et al*. 2021), as well as factors influencing neighbourhood walkability (Guliani *et al*. 2015). Emphasising the significance of examining perspectives of young people by gender regarding their local area. There is little research that integrates both subjective and objective assessments of neighbourhoods among young people, an important avenue for investigation since both the way people subjectively perceive their local neighbourhood and the objective characteristics of that neighbourhood can influence their overall health. For adults, studies have shown that those who report higher levels of neighbourhood incivilities and crime also tend to report elevated levels of anxiety and depression (Leslie and Cerin 2008, Ellaway *et al*. 2009). However, collecting subjective perceptions of local areas across a large geographical footprint with precise spatial locations can be challenging, especially among young people, and there is limited existing research in this area. Therefore, providing further evidence between subjective and objective neighbourhood assessments in the context of young people will provide valuable insights to inform the development of healthier places.

The aim of this study is to gather and investigate the subjective assessment provided by young individuals regarding their local neighbourhood. We explore potential variations in assessments based on factors such as gender, urbanicity, and area-level deprivation. Finally, this research aims to complement these subjective assessments with objective data pertaining to the accessibility of local amenities, particularly those relevant to the principles of the 20-minute neighbourhood policy.

### Research aims

The three main aims of this project were:

1. To describe young people’s perceptions of their local neighbourhood using the Place Standard Tool for Young People (PSTYP).
2. To explore whether local neighbourhood perceptions differ by a number of covariates including gender, urbanicity and area-level deprivation.
3. To explore whether there is an association between young people’s perceptions and objective assessment of the local area.

## Methods

### Participants

Young people attending secondary schools in Scotland (covering ages 12 to 18 years) that were members of the Schools Health and Wellbeing Improvement Research Network (SHINE) (https://shine.sphsu.gla.ac.uk/) were invited to participate in the study in September 2022. All SHINE schools (*n* = 160) were sent an invitation email providing information about the study; sixteen schools participated in the study, including those located in large urban areas and small rural settlements or remote islands. Participating schools were asked to recruit young people during a pre-prepared activity. The activity consisted of a 2-minute video shown during a lesson in School describing the 20-minute neighbourhood policy and its application in Scotland, at the end of the video young people then complete an online version of the PSTYP. Data were collected during October and November 2022. Scotland set out in the Government Programme for Scotland 2020-2021 and National Planning Framework: position statement to apply the concept of 20 minute neighbourhoods across the whole country (Scottish Government 2020a, 2020b), therefore we recruited a national sample.

#### Ethical considerations

Ethical approval was granted by the ethics committee of the College of Medical, Veterinary and Life Sciences (MVLS) at the University of Glasgow [Ref: 200210203].

### Subjective data - young people’s views about their local neighbourhood

Like the original Place Standard Tool (Public Health Scotland 2021), the PSTYP asked young people a series of questions about their local area, to which they had the option of responding on a scale from 1 (terrible) to 7 (excellent). Table 1 outlines the specific questions and the item name as they will be referred to in this paper. The questions from the PSTYP were slightly adapted to better align with objective data relating to the 20-minute neighbourhood dataset. Sample characteristics were also collected at the beginning of the survey; gender and postcode (postcodes are the smallest plotted areal unit in Scotland and on average there are 15 delivery points per postcode (range 1-100) (National Records of Scotland 2013)) via a free text box and age was requested via multiple choice with options for ages 12 to 18 years.

**Table 1.** Place standard tool for young people’s questions and themes.

Themes	Question
Walking, wheeling, or cycling	Can I easily walk, wheel, or cycle and move around my local area?
Public transport	How easy is it to get to where I need to or want to go by bus, train, or other forms of public transport?
Walking, wheeling, or cycling to school	Can I easily walk, wheel, or cycle to my school, college, or workplace?
Streets, squares, and buildings	What are the streets, squares and buildings like in my place?
Play	What are the spaces for play, recreation and sport like in and around my local area? For example, sports fields, gyms, skate parks and swimming pools.
Nature	Can I regularly see and visit nature (green space, hills, and quiet places)?
Meeting and talking with people	Are there a range of spaces and opportunities to meet and talk to each other face to face?
Access to services	What is your area like for accessing services you need? For example, shops, libraries, health entries and cafes?
Feeling proud and a part of my place	How proud do you feel about your local area?

### Objective data – spatial data describing presence or absence of amenities within the local neighbourhood

Objective data were obtained from two sources. Firstly, data was obtained from a previous research study that described the presence or absence of 10 key neighbourhood amenities within 800 metre (m) catchments of all Scottish residential postcodes (Olsen *et al*. 2022), providing a direct assessment of the Scottish Government’s desire to implement 20-minute neighbourhoods nationwide (Scottish Government 2023). Briefly, the postcode catchments were calculated using road and path network buffers (within the Network Analyst extension of ArcGIS Pro v2.9.2) for the following ten domains: healthy food retail, public transport, primary health care, community health resources, education, financial, accessible public open spaces, eating establishments, social and cultural locations, and recreational, sports pitches and facilities. Further information is available within Olsen *et al*. (2022).

The second source of objective data described the recorded crime rate for selected crime types per 10,000 population for all Scottish data zones from the crime domain of the 2020 Scottish Index of Multiple Deprivation (SIMD) (Scottish Government 2020c) provided as crime level quintiles (most crime prone to least crime prone) and are based on the following reported crimes provided by Police Scotland: violence, common and sexual assault, burglary, vandalism and drug crime. Data zones are small areal units used in the production of official statistics in Scotland and contain populations of between 500 and 1000 household residents (Scottish Government 2006).

#### Data processing

##### Gender

Young people were asked the question ‘Please provide your gender’ with a free text box to provide their response. Responses were coded as male or female based on their responses. Henceforth, when we describe gender, we are specifically addressing it as a social construct, distinct from sex, which pertains to biological characteristic. Gender data was collected to apply a gender-based lens on subjective assessments of place by young people. A number of young people (*n* < 10) identified as non-binary, the non-binary sample was too small for any meaningful statistical analysis and as the sample was less than 10, we could not identify these young people as per ethical requirements to avoid potential disclosable information. From this point forward, we will describe gender in terms of males and females. Invalid responses were removed.

##### Postcode

As postcode was also provided through means of free text, these had to be cleaned individually. The objective data is observed at postcode level and as a result, any partial postcodes could not be linked to this objective data. 78 young people had invalid or partial postcodes and were excluded from the subjective vs objective analysis.

In total, 399 responses were originally recorded. Responses were removed if a postcode was not provided (n: 78) and all survey questions were not answered (i.e. they started the survey but did not provide responses to questions) (n: 34). The final sample for analysis was 287; 251 provided their gender, a full postcode and were available for analysis and linking to objective data.

### Spatial data linkage

Objective data describing presence or absence of amenities within the local neighbourhood was linked to the PSTYP survey data using the postcode as the unique identifier. For each young person, the objective data contained a column for each neighbourhood amenity and a corresponding Yes/No for each, depicting presence/absence to each amenity within a 10-minute walk respectively.

The young person’s recorded postcodes were linked to the data zones they were within, to link crime, urbanicity and area-level deprivation. For each data zone, the corresponding Scottish Government two-fold urban-rural classification was obtained (Scottish Government 2018b), where settlements are considered to be rural if they inhabit less than 3000 residents, and the SIMD income domain (Percentage and number of people who are income deprived) (Scottish Government 2020c), which was chosen as a proxy measure for area-level deprivation since the overall SIMD includes measures of services accessibility account, a key interest of this study.

### Descriptive analysis

We describe the responses to the PSTYP by gender, area-level deprivation and urbanicity. Due to small numbers across the seven PSTYP responses (ranging from 1 (terrible) to 7 (excellent)), these were recategorized into three categories: ‘unsatisfied’ (scores 1–3), ‘neutral’ (score 4) and ‘satisfied’ (scores 5–7). Similar categories have been used in studies using the Place Standard tool (Kleopa *et al*. 2022). Age was recategorized into two groups, where ages 12–15 were considered ‘junior’ and ages 16–18 considered ‘senior’, which is reflective of a typical Scottish secondary school, where the school is split into two sections. Only 4.2% (n:12) of the sample were in the senior age group, meaning subsequent statistical analysis by age group was unsuitable.

**Table 2.** Corresponding subjective and objective area-level variables (from Olsen *et al*. (2022)) and source.

Subjective Domain from Place Standard Tool for Young People	Question from Place Standard Tool for Young People	Corresponding objective data	Brief description of Objective Domain	Source
Public Transport	“How easy is it to get where I need or want to go by bus, train or other forms of public transport?”	Public Transport	Any public transport stop (Bus, train, tram, underground)	Point of interest (Dec 2021) (Ordnance Survey), (Ordnance Survey 2021b)
		Frequent Public Transport	Public transport with five or more stops an hour (within 6am–9pm).	Traveline National Dataset (TNDS), (UK Government Digital Service 2015)
Play	“What are the spaces for play, recreation and sport like in and around my local area? For example, sports fields, gyms, skate parks and swimming pools”.	Recreational, sports pitches and facilities.	Gymnasiums, sports halls, and leisure centres; Athletics facilities; Golf ranges, courses, and clubs; Sports grounds, stadia, and pitches; swimming pools; and tennis facilities	Point of interest (Dec 2021) (Ordnance Survey 2021b)
Nature	“Can I regularly see and visit nature (Green space, hills and quiet places)?”	Accessible public open spaces	Public Park or Garden access points; Playing field access points; Play space access points.	Open Greenspace (Ordnance Survey 2021a)
Access to Services	“What is your area like for accessing services you need? For example, shops, libraries, health entries and cafes?”	Healthy food retail	Large supermarket (for example, ASDA); Medium sized supermarket chains (for example Tesco Metro, Sainsbury’s Local).	Point of interest (Dec 2021) (Ordnance Survey 2021b)
		Eating establishments	Restaurant or café (non-fast-food)	
		Primary Health Care	General Practitioner (GP) or NHS walk-in centre.	
		Community health resources	Pharmacy	
		Financial	Post office, bank, or cash machine (ATM).	
		Social and cultural locations	Gallery; Historic buildings; Museum; Theatre; Cinema; Social clubs; Library; and Places of worship.	
Feeling safe	“How safe and/or comfortable do I feel in my place?”	SIMD crime sub-domain	Recorded crime rate per 10,000 population.	Scottish Index of Multiple Deprivation 2020 (Scottish Government 2020c).

**Table 3.** Sample characteristics and sociodemographic profile.

Variable		Number of Young People	%
Gender	Male	121	42.2
	Female	130	45.3
	Missing	36	12.5
Age	Range: 12 to 18 years; Median 12 years.		
	Missing	<10	<10.0
Urban-Rural	Urban Areas	231	80.5
	Rural Areas	56	19.5
Area-level deprivation	Most deprived	92	32.1
	Least deprived	195	67.9
Area-level crime	Most crime prone	96	33.4
	Least crime prone	191	66.6

*Numbers below 10 suppressed across all categories to avoid potential disclosable information.

Due to small sample sizes within area-level deprivation and crime level quintiles, both were subsequently dichotomised into the most deprived/crime (quintiles 1-3) and least deprived/crime (quintiles 4,5) areas.

Chi-square or Fishers Exact Test (depending on sample size for valid test) were used to formally test whether young people’s perceptions of their neighbourhood were related to these covariates. All statistical analysis were conducted in R studio, version 4.0.3.

### Statistical analysis

Multinomial logistic regression was used to examine the relationships between young people’s perceptions of their local area and gender area-level deprivation and urbanicity. For the seven PSTYP items, ‘satisfied’ was chosen as the reference level and logit equations fitted by the model predicted log-odds of: (1) Being unsatisfied versus satisfied and (2) Feeling neutral versus satisfied. Model coefficients with corresponding p-values less than the significance threshold (*p* < 0.05) were inferred as statistically significant. Initially all covariates were included in the model for each place standard item. The least significant variable was removed one at a time using a backwards selection process, the final preferred model was chosen using Akaike Information Criteria (AIC), where the lowest AIC indicated a better-fitted model. Deviance was used to check the goodness-of-fit of the preferred models against the null model. The variables included in the final models can be seen in Table 5.

**Table 4.** Percentage (%) of Place Standard Tool for Young People item responses by unsatisfied, neutral or satisfied.

*Place Standard Tool for Young People* Items	Frequency of categorised responses (%)		
	Unsatisfied (Score 1 to 3)	Neutral (Score 4)	Satisfied (Score 5 to 7)
Walking, Wheeling, or Cycling	11.2	13.3	75.5
Public Transport	14.9	21.4	63.7
Walking, wheeling, or cycling to school	33.5	15.9	50.6
Streets, squares, and buildings	15.6	29.9	54.5
Play	28.2	22.4	49.4
Nature	12.8	11.1	76.1
Meeting and talking with people	17.7	17.7	64.5
Access to services	27.5	17.4	55.0
Feeling proud and a part of my place	11.1	20.9	68.0
Feeling safe	8.5	16.9	74.6
Fixed, cleaned, and managed	16.9	19.0	64.0

**Table 5.** Probability (Odds Ratio) of feeling unsatisfied in Place Standard Tool for Young People item perceptions of local area, by gender, area-level deprivation and urbanicity.

Place Standard Tool for Young People Items (unsatisfied vs satisfied)	Gender				Area-level deprivation				Urbanicity			
	Female (ref: male)				Most deprived (ref: least deprived)				Rural (ref: urban)			
	OR	LL 95% CI	UL 95% CI	*P* value	OR	LL 95% CI	UL 95% CI	*P* value	OR	LL 95% CI	UL 95% CI	*P* value
Walking, Wheeling, or Cycling	3.95	1.34	11.62	0.01	*				3.76	1.28	11.04	0.02
Public Transport	0.96	0.38	2.45	0.94	1.23	0.39	3.84	0.72	2.91	1.10	7.68	0.03
Walking, wheeling, or cycling to school	0.56	0.22	1.39	0.21	1.87	0.64	5.45	0.25	5.79	1.81	18.53	<0.001
Streets, squares, and buildings	1.23	0.52	2.93	0.64	2.27	0.92	5.64	0.08	3.22	1.18	8.74	0.02
Play	1.39	0.62	3.08	0.42	1.76	0.73	4.27	0.21	3.50	1.40	8.72	0.01
Nature	0.60	0.19	1.92	0.39	0.68	0.20	2.26	0.53	0.27	0.06	1.26	0.10
Meeting and talking with people	1.62	0.63	4.20	0.32	*				3.25	1.17	9.04	0.02
Access to services	1.34	0.56	3.25	0.51					2.85	1.06	7.69	0.04
Feeling proud and a part of my place	1.82	0.62	5.33	0.28	5.37	1.70	16.98	<0.001	1.23	0.39	3.87	0.73
Feeling safe	0.88	0.26	2.96	0.84	3.14	0.91	10.84	0.07	*			
Fixed, cleaned, and managed	1.62	0.53	4.89	0.39	2.69	0.79	9.14	0.11	1.88	0.51	6.99	0.34

*Insignificant variables removed from the final model using the backward selection criteria, final model chosen based on AIC.

#### Subjective vs objective – linking young people’s perception of the local neighbourhood with spatial data describing presence or absence of amenities within the local neighbourhood

The subjective views of young people were compared with corresponding objective data. In total, 5 items from the PSTYP were linked to a corresponding objective variable. Three subjective items could not be linked to an objective measure, for example, ‘feeling proud and part of place’ is a measure that is difficult to measured objectively using facility presence/absence data. Table 2 shows which questions from the survey that were appropriately linked to an objective domain, and a brief description of the objective domain data.

#### Subjective vs objective – formal analysis of young people’s perception of the local neighbourhood with spatial data describing presence or absence of amenities within the local neighbourhood

Multinomial logistic regression models were used to examine whether young people’s subjective perceptions of their local neighbourhood (dependent variable) were associated with objective data describing amenity access (Yes/No) or data zone crime rate (independent variable) within the local area.

For comparisons with more than one objective variable (i.e. public transport and access to services), initially all variables were included in the model. The least significant variable was removed one at a time and the final preferred model was chosen using AIC and goodness of fit confirmed using deviance. Variables included in the final models can be found in Table 5. Interaction terms for gender, urbanicity and area-level deprivation were run for each of the subjective and objective comparisons. Like the previous models, variables were removed one at a time by order of least significance, and the preferred model chosen using AIC.

## Results

### Participant characteristics

A total of 287 young people provided valid responses and were included in the final analytical sample, their characteristics and sociodemographic profile are described in Table 3.

There was a similar proportion of males and females in the sample (42% male vs 45% female), with 36 (12%) missing responses. The majority of young people (80.5%) lived in an urban area, which is reflective of Scotland’s urban-rural makeup (83% of the population reside in urban areas (Registers of Scotland 2023). 68% of the young people resided in the least income deprived areas of Scotland, 32% residing in the most income deprived areas. Around 67% of the sample lived in the least crime prone areas, with less than 34% in the most crime prone areas.

### Young people’s perceptions of their local area

Overall, young people reported they perceived their neighbourhood amenities well, with an average satisfaction rate of 63% for any given neighbourhood feature (Table 4). Young people reported being most satisfied with nature, with 76% of young people being satisfied with their available green space. This was followed by walking, wheeling, or cycling, where 76% reported being satisfied with means of active transportation around their neighbourhood. Over 60% reported being satisfied with public transport, meeting and talking with people, having fixed, cleaned, and managed spaces, as well as feeling proud and part of their place. Over 50% reported feeling satisfied with their streets and buildings, travelling to school, as well as access to services they need such as schools, libraries, healthcare, and cafes. Just under half (49%) of young people were satisfied with spaces for play in their area. The domain identified by young people as being most unsatisfied with was walking wheeling or cycling to school; a third (34%) reported being unsatisfied. This was followed by play (28%) and access to services (supermarkets and eating establishments) (28%).

### Perceptions by gender, area-level deprivation and urbanicity

Table 5 presents the outcome of the multinomial logistic regression models (as Odds Ratios (ORs)) to explore differences in young people reporting being unsatisfied (vs satisfied) for each PSTYP item by gender, area-level deprivation and urbanicity. Supplementary Table S1 presents the results of young people reporting feeling neutral (vs satisfied).

For gender, females were almost 4 times as likely to feel unsatisfied with walking, wheeling, or cycling around their neighbourhood than males (OR: 3.95, 95% CI: 1.34 to 11.62, p:0.012).

Young people residing within the most deprived areas were found to be over 5 times more likely to feel unsatisfied with ‘feeling proud and part of my place’(vs satisfied) than young people from the least deprived areas (OR: 5.36, 95% CI:1.70 to 16.98, p:0.004).

The most variation was seen in responses when grouped by urban-rural classification, young people residing in rural areas were 3.76 times more likely to be unsatisfied (vs satisfied) with walking, wheeling, or cycling around their local area compared to those from urban areas (OR: 3.76, 95% CI: 1.28 to 11.04, p:0.02), and were 71% more likely to feel neutral (vs satisfied) than their urban counterparts. Compared to young people residing in urban areas, young people from rural areas were more likely to feel unsatisfied with almost all aspects of their local area compared to urban residents except for feeling proud and a part of my place and fixed, cleaned, and managed (Table 5).

#### Subjective vs objective – formal analysis of young people’s perception of the local neighbourhood with spatial data describing presence or absence of amenities within the local neighbourhood

**Figure 1.**
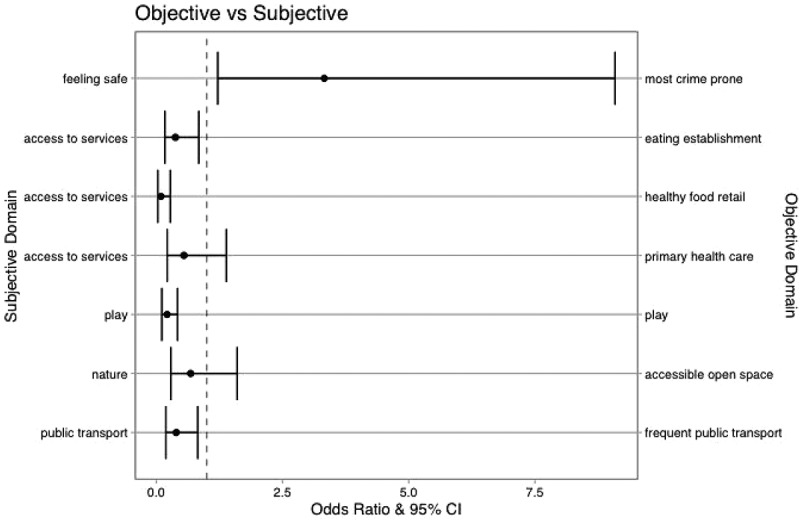
Probability (Odds Ratio) of reporting being unsatisfied with Place Standard Tool for Young People item and objective assessment of amenity presence in local area or residing in most crime prone area.

Note: If Odds Ratio <1, young person is less likely to report being unsatisfied than satisfied with the Place Standard Tool for Young People item if the amenity is present in their local area.

We found varied agreement between young people’s perception of their local neighbourhood and the presence/absence of local amenities. Figure 1 presents the plots of the odds ratios and 95% confidence intervals for young people’s subjective assessment of being *unsatisfied* with each PSTYP item and objective assessment of amenity presence in local area. Full model outputs are shown in Supplementary Table’s S2 and S3. Supplementary Figure S1 presents young people’s subjective assessment of being *neutral* with each PSTYP item and objective assessment of amenity presence in local area.

##### Public transport

There was weak agreement with access to any public transport stop but strong agreement with access to frequent public transport. Those with frequent public transport were over two times more likely to report being satisfied than those without compared to both unsatisfied (OR: 2.52, 95% CI: 1.22 to 5.22, p:0.01) and neutral (OR: 2.06, 95% CI: 1.09 to 3.89, p:0.03).

##### Nature

There was no statistically significant agreement with objective and subjective access to green space (Figure 1).

##### Play

While no statistically significant association was found between feeling neutral versus feeling satisfied with spaces for play and access to recreational, sports pitches and facilities, those with access to spaces for play were over four times more likely to report being satisfied versus unsatisfied with play-space (OR: 4.62, 95% CI:2.36 to 9.02, *p* < 0.001) (Figure 1).

##### Access to services

Variation was found in agreement between subjective and objective access to services. There were no associations found between perception and access to financial or social and cultural locations. There was agreement found between reporting satisfaction with local services and access to healthy food retail, primary health care and eating establishments. Young people were more likely to report being satisfied (vs unsatisfied) with accessing services they needed if they had access to a medium or large supermarket (OR: 10.42, 95%CI: 3.57 to 30.42, *p* < 0.001) and any eating establishment (OR: 2.63, 95% CI: 1.19 to 5.83, p: 0.02) (Figure 1).

##### Feeling safe

Agreement was found between feeling safe and levels of crime. Those living in the most crime prone areas were over three times more likely to report feeling unsafe than those in the least crime prone areas (OR: 3.33, 95% CI: 1.22 to 9.09, p: 0.019) (Figure 1).

##### Interaction terms (gender, urbanicity and deprivation)

We found interaction effects by females, who were more likely to report agreement between subjective and objective neighbourhood assessments for frequent public transport, nature and recreation (Table 6). We did not find differences in reporting of subjective vs objective neighbourhood assessments for participants living in urban/rural areas or by area-level deprivation.

**Table 6.** Interaction terms for reporting being unsatisfied with Place Standard Tool for Young People item and objective assessment of amenity presence in local area or residing in most crime prone area by gender, urbanicity and area-level deprivation.

Objective amenity present (ref: not present)	Gender: (ref:male) & Area-level deprivation (ref: least deprived) & Urbanicity (ref: urban)	Place Standard Tool for Young People Item																			
		Public Transport				Nature				Access to services				Play				Feeling Safe			
		OR	LL 95% CI	UL 95% CI	*P* value	OR	LL 95% CI	UL 95% CI	*P* value	OR	LL 95% CI	UL 95% CI	*P* value	OR	LL 95% CI	UL 95% CI	*P* value	OR	LL 95% CI	UL 95% CI	*P* value
Frequent public transport	Female	**6.83**	**1.14**	**40.80**	**0.035**																
	Rural	0.46	0.07	3.07	0.42																
	Most Deprived	*																			
Open accessible public spaces	Female					**7.75**	**1.06**	**56.54**	**0.04**												
	Rural					*															
	Most Deprived					<0.0001	0	Inf	0.99												
Healthy food retail	Female									0.68	0.06	7.41	0.74								
	Rural									*											
	Most Deprived									1.43	0.08	26.85	0.81								
Primary health care	Female									0.55	0.07	4.15	0.56								
	Rural									*											
	Most Deprived									1.21	0.11	13.61	0.87								
Eating establishments	Female									3.35	0.58	19.32	0.17								
	Rural																				
	Most Deprived									0.23	0.02	2.85	0.25								
Recreational, sports pitches and facilities	Female													**6.12**	**1.18**	**31.66**	**0.03**				
	Rural													0.45	0.07	3.04	0.41				
	Most Deprived													0.29	0.04	2.29	0.24				
SIMD crime statistics	Female																	0.96	0.10	9.23	0.97
	Rural																	0.32	0.02	5.87	0.04
	Most Deprived																	0.04	0.03	5.54	0.52

*Insignificant variables removed from the final model using the backward selection criteria, final model chosen based on AIC.

## Discussion

This first aim of this study was to describe young people’s perceptions of their local neighbourhood using a validated instrument and explore variation in responses by gender, urbanicity and area-level deprivation. Using the Place Standard Tool for Young People, we found that the majority of young people reported being satisfied with their local area, believing they were well-placed and satisfied with many of the every-day aspects of their neighbourhood. In general, young people reported being most satisfied with nature and walking, wheeling, or cycling around the area, and least satisfied with walking, wheeling, or cycling to school. We found variation in young people’s reported perception of their local area by gender, area-level deprivation, and urbanicity.

The second aim of the study was to compare young people’s subjective assessment of their local area with objective data describing the presence/absence of a number of key local amenities and the area-level crime rate. We found agreement between young people’s reporting being satisfied with their local area and objective data describing frequent public transport, spaces for play and recreation, and access to services (healthy food retail and eating establishments). We also found agreement between young people reporting feeling unsafe and area-level crime rates.

### Comparison with other literature

Incorporating the perspectives of young individuals regarding their neighbourhood, along with examining the correlation with objective attributes, is crucial to comprehending what constitutes a high-quality and health-promoting environment for them. The challenge lies in developing and implementing place-based interventions that can tangibly and reliably impact health outcomes. It is important to note that simply having more amenities does not necessarily translate to increased usage, and a higher footfall within places may not uniformly result in health benefits across demographic groups, as observed in previous research (Audrey and Batista-Ferrer 2015). While local environments play a significant role in the lives of young people, evidence from small qualitative studies has indicated that access to health-promoting resources may not be equitable across different sociodemographic groups (Eriksson and Dahlblom 2020). Our study contributes valuable evidence by demonstrating that the presence of amenities is associated with positive perceptions among young people, based on a larger sample size. This finding supports the identification of targeted neighbourhood intervention areas that are underserviced, particularly in terms of positive perceptions of having frequent public transport, recreational services, and health food retailers. Additionally, our study underscores the link between perceptions of crime and area-level crime variables. Existing evidence strongly suggests that residing in high-crime areas is negatively associated with health outcomes (Visser *et al*. 2021), and our study emphasises that young people perceive this reality. Therefore, intervention strategies are essential to mitigate against the negative health impacts stemming from such perceptions.

We found that young people reported being least satisfied with walking, wheeling or cycling to school, despite higher reported levels of satisfaction for walking, wheeling or cycling in their local neighbourhood. This contrasting finding highlights that different factors about the local environment may influence young people’s ability to walk, wheel or cycle for leisure purposes as opposed to travelling to school. Factors such as traffic, attractive routes, ability to chat to friends, time constrains, and parental perceptions of route safety have been associated with young people’s active transportation to school (Mandic *et al*. 2015). Distance to school is also a key factor in the likelihood of walking, wheeling or cycling to school, where previous studies have shown a significant decrease from 64% to 18% in reported active travel to school when the home-to-school distance is over 2.25 km (compared to less than 2.25 km) (Mandic *et al*. 2023). Although for this study we were unable to assess home-to-school distance, data from the 2011 Scottish Census showed that over 60% of young people (aged 12 to 17 years) resided over 2 km from their school and this could be an important barrier for many young people in Scotland (Transport Scotland 2016).

Young people living in rural areas of Scotland reported being more dissatisfied with their local neighbourhood compared to those within urban areas. Rural areas, although greener, are often less walkable than urban areas, with poorer pedestrian infrastructure whereas urban areas are generally more walkable (Watson *et al*. 2020, Bucko *et al*. 2021). Access to a range of services, amenities and public transport within rural areas is poor (Olsen *et al*. 2022), and our results emphasise this from young people’s subjective assessment of their local neighbourhoods. It is important that the needs of young people living in rural areas are considered in future place-based planning; the National Youth Agency (2021) highlighted that there is none or relatively little youth provision in rural areas, leading them vulnerable to isolation, loneliness and poor mental health.

Overall, young people’s perceptions of their local area were similar for males and females, with most domains showing no significant differences. However, females were nearly 4 times more likely to feel unsatisfied with walking, wheeling, or cycling around their neighbourhood than males. There is a large global evidence base highlighting that, for young people, females participate less in physical activity than males (Timperio et al. 2004, Althoff et al. 2017), measured as sex. Studies of young people have shown sex-based difference in their use, highlighting that amenities for walking, wheeling, and cycling in UK public parks were predominantly utilised by males, with females expressing a sense of exclusion from these spaces (Make Space For Girls 2023). The Make Space For Girls (2023) report suggests that future provisions for walking, wheeling, and cycling in parks should undergo an equality impact assessment. Likewise, cycling advocacy groups have urged for meaningful opportunities for young people to participate in decision-making processes regarding future provisions for walking, wheeling, and cycling (Sustrans 2022).

We found an association between young people’s perception of safety and levels of crime in their neighbourhood; young people living in the most crime-prone areas were greater than 3 times more likely to feel unsafe. Given the importance of perceived neighbourhood safety in that feeling unsafe can induce social isolation, adverse mental health outcomes and limits freedom and physical activity (Aneshensel and Sucoff 1996, Weir *et al*. 2006), the literature from an adolescent perspective of what constitutes a safe neighbourhood is limited.

Young people residing in the most deprived areas were more likely to be unsatisfied with their local area. This association may be attributed to various factors, such as elevated levels of street-level incivilities, which have been reported as being worse in deprived areas (Chong *et al*. 2013). Prior studies have highlighted that young people are sensitive to the aesthetics of their local neighbourhoods, and their use of these environments can be hindered by issues like litter and vandalism (Day and Wager 2010). This observation is important as it highlights that disparities in perceptions of local environments among young individuals exist between those living in the most and least deprived areas. It is important to tackle neighbourhood incivilities as they have been linked to worse health outcomes in adults (Dunstan *et al*. 2013) and could lead to widening of health inequalities.

We observed varying associations between subjective and objective measures when assessing different amenities. Specifically, there was agreement between access to amenities and eating establishments, as well as healthy food retail. However, there was a lack of agreement between access to amenities and primary health care, pharmacies, financial amenities, and social and cultural locations. This difference suggests that retail amenities might hold greater significance within a local neighbourhood for young people compared to others. Further research is required to understand the amenities within local areas young people require for their day-to-day needs.

Our study did not reveal any alignment between satisfaction with nature and actual presence of it. This aligns with prior research conducted on adults, which found no agreement between objectively measured and self-reported distances from greenspaces (Macintyre *et al*. 2008). Additionally, in subsequent research involving adults, it was noted that there was poor correspondence between perceptions of being well-situated with regard to amenities and actual presence within an 800-meter buffer (Macdonald *et al*. 2013).

### Importance for health

Perceptions of one’s local neighbourhood can directly influence health outcomes, with individuals perceiving their neighbourhood positively being less likely to report fair/poor health or emotional distress, while negative perceptions are associated with a higher likelihood of reporting chronic conditions (Wilson *et al*. 2004). In this study, we observed a correlation between young people who reported being well-situated for frequent public transport, access to play and recreation spaces, and availability of services, and their high satisfaction with these amenities. This satisfaction may have both direct and indirect impacts on health. The health benefits of utilising play and recreation spaces include increased physical activity, stress reduction, and social interaction. However, the proximity and quality of these amenities are key factors influencing use by young people (Godbey 2009). The significance of our findings lies in the association between neighbourhood proximity to these amenities and reported satisfaction, prompting the need for further research to explore whether these amenities are actively used by young people and to quantify potential health effects.

Living in areas with high crime rates was linked to feeling unsafe by young people. This finding is crucial for developing health mitigation strategies for young individuals residing in crime-prone areas. In deprived neighbourhoods characterised by high crime rates and disorder, there is an association with poor mental health and well-being outcomes for young people (Visser *et al*. 2021). Although no correlation was found between access to open spaces and reported satisfaction with these spaces, proximity to and access of such spaces can offer significant health benefits in later life, including a reduced incidence of diabetes, asthma, cardiovascular diseases, and all-cause mortality (Ige-Elegbede *et al*. 2022). Further research is necessary to understand whether young people actively utilise these spaces and to identify potential barriers limiting their health benefits.

### Strengths and limitations

There were a number of strengths to this study. A major strength of this study was the comparison of objective and subjective views of neighbourhood, for not just one amenity, but several. This study built on existing place-based research using the Place Standard Tool by comparing subjective views of neighbourhood by subgroups. This research also added to limited research using the Place Standard Tool and the version for young people and as far as we are aware, there is no such existing research that compares objective and subjective views of place for young people in the United Kingdom. Not only has the Place Standard Tool been validated for assessing neighbourhoods (Ocaña *et al*. 2022), but the young people’s version was developed and piloted with young people, ensuring the questions are relevant and tailored to their age group.

The robustness of the objective data used also added to the strengths of the study. The objective data was created using road and network path analysis, improving the spatial accuracy of the presence of amenities within walkable buffers around young people’s home locations. The objective domains used align with Scottish planning policy surrounding 20-minute neighbourhoods with highly comprehensive objective data sourced from the UK’s national mapping agency (Ordnance Survey).

The study was limited by a small sample size, the free-text boxes for gender and postcode also resulted in several invalid data that had to be removed. This also meant we could only assess differences between males/females and were unable to include subgroup analysis for other recorded genders, such as non-binary young people. Our question asked to young people in our study ‘please provide your gender’ prevented the ability to disentangle biomedical ‘sex’ differences from sociocultural ‘gender’ continuum, as we did not specify that we did not want young people to provide their biomedical sex, especially as we used a free-text box to capture this information. Although sex-based differences have been shown in the assessment of neighbourhood characteristics (for example, mental health outcomes (Gupta *et al*. 2021)), few studies have explored gender-based differences in assessments of local neighbourhoods and health, this is usually due to surveys collecting data describing the participants sex (biological characteristic). This highlights a gap and call for future research to collect data regarding gender to understand the impact of neighbourhood design on health. In practice, there has also been a call to integrate gender perspective into every stage of policy processes, from design, implementation, monitoring, and evaluation, to create safe and equitable places (Palitm 2020).

When split by age, over 73% of the sample were 12 years old, reasons for this are unclear but as the survey was sent out to schools during a crucial study period in the run up to Scottish preliminary exams, teachers may have been less likely to provide the older young people with the survey. We attempted to remedy this by aggregating the age groups together to form ‘junior’ and ‘senior’ age groups, however the senior age group included only 12 participants rendering subgroup analysis unsuitable. Participation in the study was voluntary, and only 10% of SHINE schools opted to take part, potentially influenced by time and staffing limitations within schools. Ethical considerations, ensuring student anonymity, prevented us from collecting information on the specific schools attended by participants. Consequently, we were unable to cluster our analysis or provide insights into potential biases among participating schools.

Although the survey questions were slightly adapted to align with the objective 20-minute data, we were still only able to make a few comparisons between the PSTYP items and the objective domains. This was either due to a lack of available data or no suitable objective comparison. For example, we could not compare feeling proud and a part of place with the presence or absence of an amenity. However, for walking, wheeling, cycling (to school), there was no available data on the presence or absence of cycle paths.

## Conclusions

Overall, young people rated many aspects of their local environment favourable, such as nature and walking, wheeling, or cycling. However, young people were dissatisfied with being able to walk, wheel or cycle to school, spaces for play and recreation and access to services. Females were less likely to be satisfied with being able to walk, wheel or cycle around their local area than males, highlighting a requirement for further work create places to support females in travelling safely around their local area. Young People living in rural areas were less satisfied with many aspects of their local neighbourhood, compared to those in urban areas. Targeted interventions are required in rural communities to improve young people’s satisfaction with their local neighbourhood and further research is required to understand why young people are unsatisfied with being able to walk, wheel or cycle to school.

## Supplementary Material

Supplemental Material
