# Supplementary material for: Exploring young people’s views of their local area related to the 20-minute neighbourhood policy: a national cross-sectional study
Source: Cities Health. 2024 Feb 29;8(6):1081–93. doi: 10.1080/23748834.2024.2315804 (PMC11614036; doi:10.1080/23748834.2024.2315804)
Supplement: Supplemental Material [file RCAH_A_2315804_SM6003.pdf]

Exploring young people's views of their local area related to the 20-minute neighbourhood policy: a national cross-sectional study.

Supplementary Materials

Supplementary Table 1: Probability (Odds Ratio) of feeling neutral in Place Standard Tool for Young People item perceptions of local area, by gender, area-level deprivation and urbanicity

Place Standard Tool for Young People Items (neutral vs satisfied)	Gender				Area-level deprivation				Urbanicity			
	Female (ref: male)				Most deprived (ref: least deprived)				Rural (ref: urban)			
	OR	LL 95% CI	UL 95% CI	P value	OR	LL 95% CI	UL 95% CI	P value	OR	LL 95% CI	UL 95% CI	P value
Walking, Wheeling, or Cycling	1.6	0.7	3.4	0.2					0.3	0.1	0.7	0.0
Public Transport	0.9	0.4	1.7	0.7	1.9	0.8	4.2	0.1	0.4	0.2	1.0	0.0
Walking, wheeling, or cycling to school	0.5	0.2	1.1	0.1	2.2	0.8	6.0	0.1	0.3	0.1	1.4	0.1
Streets, squares, and buildings	1.6	0.8	3.0	0.2	0.7	0.4	1.5	0.4	1.0	0.5	2.4	0.9
Play	1.5	0.7	3.0	0.3	1.2	0.5	2.6	0.6	0.5	0.2	1.4	0.2
Nature	0.6	0.2	1.5	0.3	0.6	0.2	1.5	0.3	0.7	0.2	2.0	0.5
Meeting and talking with people	1.6	0.8	3.4	0.2					0.5	0.2	1.2	0.1
Access to services	1.0	0.5	2.3	1.0					0.4	0.1	1.1	0.1
Feeling proud and a part of my place	0.9	0.4	1.8	0.7	1.7	0.7	4.0	0.3	0.5	0.2	1.1	0.1
Feeling safe	0.7	0.3	1.4	0.3	0.6	0.2	1.3	0.2				0
Fixed, cleaned, and managed	1.0	0.4	2.5	1.0	1.3	0.5	3.8	0.6	1.2	0.4	3.6	0.8

Supplementary Table 2: Probability (Odds Ratio) of reporting being unsatisfied with Place Standard Tool for Young People item and objective assessment of amenity presence in local area or residing in most crime prone area.

**Objective amenity present (ref: not present)**	**Place Standard Tool for Young People Item**																			
	**Public Transport**				**Nature**				**Access to services**				**Play**				**Feeling Safe**			
	**OR**	**LL 95% CI**	**UL 95% CI**	**P value**	**OR**	**LL 95% CI**	**UL 95% CI**	**P value**	**OR**	**LL 95% CI**	**UL 95% CI**	**P value**	**OR**	**LL 95% CI**	**UL 95% CI**	**P value**	**OR**	**LL 95% CI**	**UL 95% CI**	**P value**
Frequent public transport	0.40	0.19	0.82	0.01																
Open accessible public spaces					0.68	0.29	1.60	0.38												
Healthy food retail									0.10	0.03	0.28	<0.01								
Primary health care									0.55	0.22	1.39	0.21								
Eating establishments									0.38	0.17	0.84	0.02								
Recreational, sports pitches and facilities													0.22	0.11	0.42	<0.01				
SIMD crime statistics																	3.33	1.22	9.09	0.02

Supplementary Table 3: Probability (Odds Ratio) of reporting being neutral with Place Standard Tool for Young People item and objective assessment of amenity presence in local area or residing in most crime prone area.

**Objective amenity present (ref: not present)**	**Place Standard Tool for Young People Item**																			
	**Public Transport**				**Nature**				**Access to services**				**Play**				**Feeling Safe**			
	**OR**	**LL 95% CI**	**UL 95% CI**	**P value**	**OR**	**LL 95% CI**	**UL 95% CI**	**P value**	**OR**	**LL 95% CI**	**UL 95% CI**	**P value**	**OR**	**LL 95% CI**	**UL 95% CI**	**P value**	**OR**	**LL 95% CI**	**UL 95% CI**	**P value**
Frequent public transport	0.49	0.26	0.92	0.03																
Open accessible public spaces					0.79	0.31	2.02	0.63												
Healthy food retail									0.46	0.19	1.12	0.09								
Primary health care									0.38	0.15	0.99	0.05								
Eating establishments									0.74	0.31	1.79	0.50								
Recreational, sports pitches and facilities													0.68	0.31	1.49	0.34				
SIMD crime statistics																	1.20	0.56	2.55	0.64

Supplementary Figure 1: Probability (Odds Ratio) of reporting being neutral with Place Standard Tool for Young People item and objective assessment of amenity presence in local area or residing in most crime prone area.

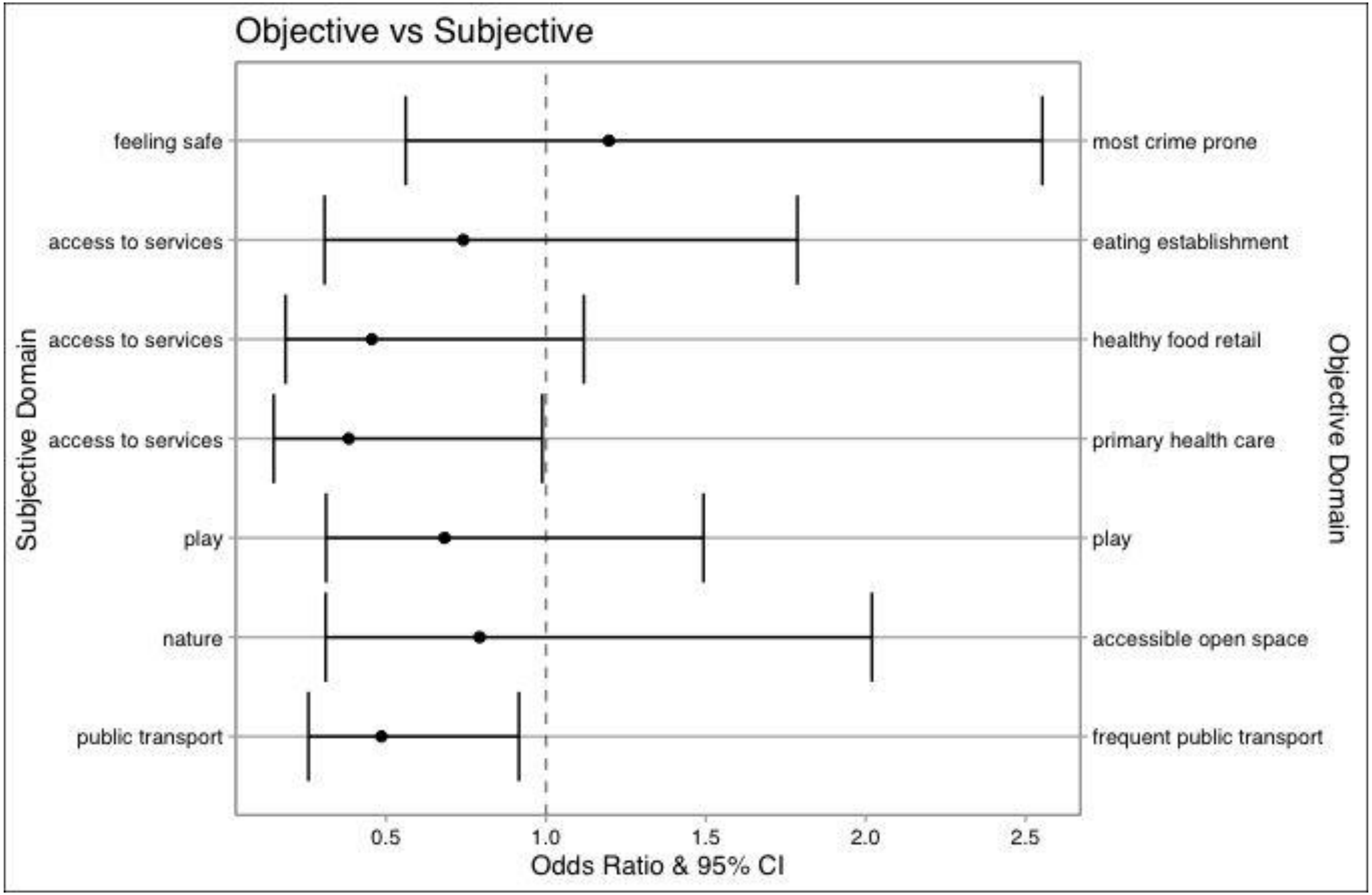

Note: If Odds Ratio <1, young person is less likely to report being neutral than satisfied with the PSTYP item if the amenity is present in their local area.
